# Supplementary material for: Parental teasing and body dissatisfaction in White and South Asian females: An exploratory cross-sectional analysis using moderated mediation
Source: PLOS Ment Health. 2025 Sep 18;2(9):e0000283. doi: 10.1371/journal.pmen.0000283 (PMC12798244; doi:10.1371/journal.pmen.0000283)
Supplement: Attachment — Submitted filename: Response to Reviewers.docx [file pmen.0000283.s002.docx]

Dear Dr. Catherine Malboeuf-Hurtubise,

We are writing in response to the reviews of our manuscript, “Parental Teasing and Body Dissatisfaction in Caucasian and South Asian Females: An Exploratory Cross-Sectional Analysis Using Moderated Mediation,” submitted to *PLOS Mental Health*.

We sincerely appreciate the insightful comments and constructive feedback provided by the reviewer. We have carefully addressed these suggestions and believe these revisions have substantially strengthened our manuscript. Below, we provide a detailed point-by-point response to each reviewer comment, outlining the specific revisions made and indicating corresponding sections and pages of the revised manuscript.

Again, we are grateful for your and the reviewer’s insightful feedback, and look forward to your response. We are happy to revise the manuscript further as needed.

**Data Availability**

The data is now publicly available at: <https://osf.io/vahrz/?view_only=1b3f6f4c9f8a4216927ac30d06345714>

**Major Comments:**

1. “*This could potentially be suitable for publication but needs a major revision to address limitations, particularly in relation to conclusions drawn based on cross-sectional data. Throughout, there are examples of language that infers causality or temporal sequence - but there is no longitudinal data to support these statements.”*

We thank the reviewer for this comment and fully agree with the importance of distinguishing correlation from causation in cross-sectional designs. We reviewed the entire manuscript and revised all language that implied causality or temporal progression. For example, phrases such as “impact of parental teasing on body dissatisfaction,” “development of BD,” or “leads to depression” were replaced with language emphasizing co-occurrence or associations (e.g., “was associated with,” “was linked to,” “showed a positive correlation”). We also revised the abstract, introduction, and discussion to include clear disclaimers that the study’s design precludes any causal conclusions.

2. *“The role of reverse causation and unmeasured confounders is ignored… There is no reason why the direction/sequence proposed is any less plausible than other hypothesized directions.”*

As mentioned above, we have revised our manuscript to clearly emphasize the exploratory, correlational, and cross-sectional nature of our analyses, carefully removing language that implies causality or temporal sequence throughout. The title, abstract, introduction, and results sections have been adjusted accordingly. Additionally, we included a new section in the Limitations section (Discussion, page 17) explicitly addressing the risk of reverse causality and unmeasured confounds. Specifically, we state: “Another important limitation is the cross-sectional design of the study, which prevents causal inferences. While the findings suggest that parental weight teasing contributes to depressive symptoms and body dissatisfaction, it is also possible that individuals who already experience greater psychological distress may be more likely to recall, interpret, or report parental comments as teasing. This raises the possibility of reverse causation, where existing symptoms influence perceptions of parental behavior. Additionally, there may be unmeasured confounding variables (such as family dynamics, parental mental health, history of trauma, or broader sociocultural stressors) that simultaneously influence both the likelihood of being teased and the development of depression or BD. These unmeasured factors may have contributed to the observed associations and should be considered in future research. Longitudinal and multi-method studies are needed to better disentangle the directionality and underlying mechanisms of these relationships.”

3. *“There was no pre-registration of the analysis plan (e.g. Open Science Framework or similar). There are predictors/exposures, outcomes/dependent variables, possible mediators [limited by cross-sectional data, they were measured at the same time point], and moderators / subgroups. There is no clear research question that positions the status of these clearly upfront. Sometimes self-esteem is treated as a moderator, sometimes a covariate, in the text. This gives the whole thing an exploratory feel, and it would be better in my view to position the manuscript as exploratory throughout….Examples of language throughout I think step beyond the cross-sectional data (not an exhaustive list): Onset, development, outcomes, correlates, emergence, subsequent, leads to, development of, impact of X on Y. This all has to be rewritten. Only in one paragraph in the discussion section, reflecting briefly on *possible* mechanisms for future longitudinal research, should this sort of conjecture appear. You have cross-sectional correlations, nothing longitudinal, and unmeasured confounders that could explain some of these (plus the problem of reverse causation).*

Thank you for this valuable input. We have revised the final paragraph of the Introduction (Page 6) to clearly highlight the exploratory nature of the study and explicitly outline the three guiding research questions. These are: 1) Do self-reported levels of parental weight-related teasing differ between Caucasian and South Asian participants? (2) Is parental teasing associated with BD, and is this relationship mediated by depressive symptoms? and (3) Does self-esteem moderate associations between teasing, depressive symptoms, and BD, consistent with a moderated mediation framework? Additionally, the “Variables” section (Page 9) has been added in accordance with STROBE guidelines to clearly define each variable’s role (e.g., exposure, mediator, moderator, or covariate). We also emphasized that no pre-registration was conducted due to the exploratory design on page 8 of the revised manuscript.

4. *“The main strength is having two samples with the same measures, to compare Caucasian and South Asian females. The figure shows that parental weight teasing has a stronger 'direct' effect to body dissatisfaction for South Asians, whereas more of the association is mediated by depression in Causasian. Direct effects in reality, are unmeasured mechanisms/mediators, so in the discussion you need to reflect more on what those might be (cautiously). The figure is clear, but doesn't need BMI - all outcomes and mediators and predictors should controlled for covariates age, bmi etc. However - adjusting for covariates that might be on the causal chain can produce biased estimates (there is an established literature in epidemiology on why we shouldn't control for covariates that are mechanisms/mediators you may want to consult), so do this with caution. To my mind BMI is a possible mediator or even reverse causation - BMI due to differences in diet and physical activity could generate parental teasing and dissatisfaction. There is no reason why that direction/sequence is any less plausible than your hypothesized direction, without longitudinal data that can test this”*

We appreciate this important statistical point. In response, we have clarified in the Statistical Approach section (Pages 9-10) indicating that BMI was included only as a covariate to adjust for potential confounding, and not as a mediator. We also explicitly noted the risks associated with controlling for variables on the causal pathway and cited relevant epidemiological literature cautioning against such practices. In the discussion (Page 17), we now outline alternative explanations, including the possibility that BMI differences may drive teasing or BD. Specifically we state: “Compounding these challenges is the role of BMI, which we included as a covariate to adjust for baseline group differences but which may itself lie on the causal pathway between lifestyle factors (e.g., diet, physical activity) and both teasing and BD. Controlling for a potential mediator, rather than a true confounder, can introduce statistical bias and distort effect estimates, a concern well documented in the epidemiological literature (Schisterman et al., 2009). Moreover, alternative causal sequences are plausible, such as higher BMI increasing the likelihood of teasing and BD, or body dissatisfaction influencing eating behaviors and subsequent changes in BMI. Without longitudinal data, we cannot determine the directionality of these relationships or rule out the possibility that teasing, depressive symptoms, BD, and BMI are interconnected in more complex or bidirectional ways.”

5. *“All scales need internal consistency measures (Cronbach’s alpha).”*

Thank you for this comment. We have addressed this by reporting Cronbach’s alpha values for all study measures in Table 2, separately for Caucasian and South Asian participants. Internal consistency was high across all scales, with alpha values ranging from .81 to .98.

6. *Far too usage/emphasis on "significance" - this is much less important than effect size (translate into clinical implications, % depressed etc, size of difference between groups) and confidence interval (reflecting precision and statistial power).*

We have revised the “Tests of Mediation” and “Tests of Moderated Mediation” sections of the manuscript to address this concern by shifting away from binary significance testing and placing greater emphasis on effect sizes, confidence intervals, and clinically meaningful interpretation. As one example, we now state: “The strongest association between teasing and depression occurred for participants with high self-esteem (B = 2.79, t(198) = 2.20, p = .029), indicating that among Caucasian females with high self-esteem, greater parental teasing was associated with higher levels of depressive symptoms.” Confidence intervals are reported for all direct and indirect effects to reflect estimate precision, and we specify sample sizes for each self-esteem subgroup (e.g., n = 29 for high self-esteem in the Caucasian sample) to contextualize the robustness of findings. P-values remain in the text only where necessary for interpreting moderation effects, but the overall presentation of results now focuses on effect size interpretation and the strength of relationships. These updates are reflected throughout the revised Results and appear on Pages 11–14.

7. *Please can the Figure be one set of results, that directly correspond to the research questions at the end of the revised introduction. If self-esteem is a significant moderator, split panels by self-esteem. So up to six panels (South Asian x Caucasian, low/med/high self-esteem). Draw out the key differences - noting that unmeasured mechanisms are stronger in South Asian (because bigger direct effect).*

We appreciate this thoughtful suggestion. In response, we have replaced Figure 1, which previously illustrated the simple mediation model, with a revised six-panel Figure that depicts the results of the moderated mediation analyses. Specifically, this updated figure presents standardized path coefficients at low, moderate, and high levels of self-esteem for both Caucasian and South Asian participants, allowing for direct visual comparison across subgroups.

We hope that this revised Figure provides a clearer and more targeted representation of the analytic model corresponding to our research questions, and highlights key differences in how self-esteem moderates the link between parental weight teasing and depression in each group. The Figure also illustrates the direct and indirect associations with body dissatisfaction, and we tentatively note that the stronger direct effect observed among South Asian participants (particularly at lower self-esteem levels) may suggest the influence of unmeasured mechanisms contributing to body dissatisfaction beyond depressive symptoms.

Additionally, the Results section has been revised to better reflect the structure and findings of the moderated mediation model now shown in Figure 1. We have also expanded our reporting of the simple mediation findings in text, given the removal of the original mediation figure.

8. *“Structure of discussion section should be improved and hypotheses/exploratory ideas limited to one paragraph.”*

Thank you for this helpful suggestion. We have revised the Discussion section to improve overall structure and clarity. Specifically, we have reorganized the content to ensure a more logical flow, moving from a summary of key findings to interpretation in light of existing literature, followed by limitations and implications. In line with your recommendation, we have consolidated exploratory ideas and hypotheses regarding cultural differences and the role of self-esteem into a single paragraph (see Discussion, Page 16). This paragraph now clearly identifies these ideas as tentative and frames them as directions for future research, rather than definitive conclusions. We hope these revisions enhance the readability and interpretive rigor of the Discussion section.

**Minor Comments:**

9. *Page 4: “The focus on Caucasian samples is partly due to 'outdated notions' but there is a more fundamental and wider issue that health research in general under-represented non-Cauasian volunteers, which must be acknowledged. Your study adds value because you are contributing data on a group where data is pauce. Focus on that strength.”*

Thank you for this important point. We revised the Introduction (Page 4) to acknowledge the broader issue of underrepresentation of non-Caucasian groups in health research. We now emphasize that the study contributes novel data to an underrepresented population (South Asian Canadian females), where research on parental teasing and BD is sparse.

10. *Page 5: “I was puzzled here because you say models linking teasing and BD may extend, but I was left thinking why would the mechanisms be different, why wouldn't we just assume they are the same for all parents/children. This is picked up later in the manuscript and needs foregrounding here to more strongly justify the departure point for your study.”*

Thank you for this thoughtful comment. In response, we revised the Introduction to more clearly foreground the rationale for examining potential differences in the mechanisms linking parental teasing and BD across cultural groups (Pages 5-6). Specifically, we expanded our discussion of cultural values, familial dynamics, and the sociocultural context of South Asian families living in Westernized countries. These additions help clarify why it may not be appropriate to assume that teasing operates in the same way across all parent–child relationships, and why cultural factors might moderate the meaning or psychological impact of parental weight teasing. We also highlight relevant literature suggesting that the emotional salience of familial interactions may be amplified within South Asian communities due to intergenerational and cultural pressures.

11. *Page 5: “Why two types - weight, competence. What happened to competence later in the manuscript? This is not clear - competence is dropped from the figure (again, this adds to the exploratory feel of the paper).”*

In the revised Materials section (Page 8), we explain that competence teasing was originally measured but excluded from analyses due to its weaker theoretical relevance to BD. This was determined a priori based on literature identifying weight teasing as more central to body dissatisfaction.

12. *Page 5: “Do not say 'the discrete mechanisms' - you picked two, why? And in the figure, it's clear these weren't the strongest mechanisms in South Asian groups (the direct effect means there are unmeasured mechanisms open for future research).”*

Thank you, we have removed the phrase “discrete mechanisms” from the Introduction and replaced it with specific variable roles. Each variable (depression, self-esteem, BMI) is now clearly defined in the Variables section (Page 9).

13. *Page 5: “A problem here is you list depressive symptoms, self-esteem, and BMI as mechanisms - but self-esteem becomes a moderator later! This is very confusing for reviewers and readers. You need to more carefully position the status of your variables, pre-register these, and connect to more precise research questions…The sentences 'This research enhances....' onwards do not belong at the end of the introduction. The precise research questions should be here, including proposed moderators / subgroups (see STROBE equator guidelines and checklist).”*

You are absolutely correct and we appreciate you pointing this out. We have explicitly clarified in the revised Introduction and Variables section that self-esteem serves as a moderator of the teasing-depression association, eliminating ambiguity regarding its role (see revised Variables section on page 9 of the revised manuscript).

14. *Page 7: “Assuming participants were anonymised? Add for clarity. There is no information on recruitment - where are they recruited from and what was the criteria? See STROBE”*

We revised the Participants and Procedure sections (Pages 7-8) to clarify that participants provided informed consent, were assured anonymity, and were recruited from an undergraduate psychology course via a research participation system. Inclusion criteria are provided within the Participants section of the revised manuscript.

15. *Page 8: “Do you mean assumptions of collineairty were met, or there was no evidence of multicollinearity?”*

We revised the text in Results to clarify that there was “no evidence of problematic multicollinearity,” explicitly addressing the reviewer’s comment (Page 11).

16. *Page 8: “Why are CIs shown but no point estimate? Very odd.”*

You are correct, we have now included point estimates alongside the confidence intervals for the indirect paths in the Mediation section (Pages 11-12), as generated by the PROCESS macro. This correction was made to ensure that confidence intervals were not presented in isolation.

17. *Page 9: “This section is much too complicated and you didn't clearly specify moderated mediation in your introduction ending. Keep it simple - if you hypothesis is that the mediation depends on a moderator, test whether a model with the interaction term fits significantly better than one without (significance is OK for that sort of test, but not for main analysis results). If it needs the interaction, split into two groups and report separately. Clarify what status self-esteem has - I did not understand this, so readers may not either. Stop saying "significant" and explain in simple terms what the effect sizes mean in practice in absolute terms.”*

Thank you for this valuable input. We have substantially simplified the moderated mediation section, clearly stating self-esteem’s moderation of the teasing-to-depression path and explicitly defining subgroups (high/moderate/low self-esteem), following reviewer recommendations (see revised Tests of Moderated Mediation on pages 12-13).

18. *Page 10: “The focus on self-esteem here is odd because the Figure, which typically shows the main results / primary research question, doesn't feature self-esteem at all.”*

Thank you for this helpful observation. In response, we have removed the original simple mediation figure and replaced it with a new six-panel figure that features self-esteem as a moderator, aligning the visual representation with our primary research questions. We hope this updated figure more clearly reflects the role of self-esteem in the moderated mediation model and enhances the clarity of the study’s main findings.

19. *Page 11: “The sentence 'our findings provide support for an emerging...' needs rewriting, it is not clear.”*

Thank you, we have replaced the sentence in question with the following: “These findings contribute to the growing body of literature that challenges the notion that BD is primarily a concern among Caucasian females. Although BD has traditionally been viewed as most prevalent in this group (Holmqvist & Frisén, 2010), emerging research suggests that non-Caucasian females may experience similar or even greater levels of BD (Gupta & Gupta, 2001; Jung & Forbes, 2007; Lipinski & Pope, 2002).” (Pages 15-16).

20. *Page 12: “Here, it is very interesting to read that cultural beauty standards may be contradictory and more complex for South Asian compared to Cauasian females. For someone who doesn't know this, more detail is needed - and isn't this the departure point for your research question i.e. the motivation for the hypotheses? It should be up front in the introduction section. Why would we expect the models to differ - because of these nuances. Make this more explicit in a revision.”*

Thank you, this section of the Discussion was revised in full, given your comments in point #8. The revised section can now be found on Page 16.

21. *Page 13: “'statistical factors' is the wrong phrase here because it may be confused with 'factors' in factor analysis.”*

You are absolutely correct, we have replaced the term with “variables”.

22. *Page 14: “Reverse causation! Must be acknowledged - dietary factors and physical activity differences may influence BMI leading to depression, teasing etc. The teasing may not be cultural differences, but similar responses to BMI, driven by lifestyle differences. Of course it may be bidirectional, but that's the limitation of cross-sectional data - we don't know.”*

Thank you, as previously noted in point #2 above, this section has been revised to address the concerns listed here (pages 17-18).

We thank the editor and reviewer again for their thoughtful and constructive feedback. We believe the revised manuscript better reflects the study’s exploratory nature, cultural significance, and methodological rigor. We would be happy to further revise the manuscript should additional recommendations arise

Sincerely,

Amané Halicki-Asakawa, Dilpreet Lidder, and Maya Libben

Psychopathology, Lifespan, and Neuropsychology Lab

University of British Columbia Okanagan

Kelowna, BC, Canada
