# Supplementary material for: Parental teasing and body dissatisfaction in White and South Asian females: An exploratory cross-sectional analysis using moderated mediation
Source: PLOS Ment Health. 2025 Sep 18;2(9):e0000283. doi: 10.1371/journal.pmen.0000283 (PMC12798244; doi:10.1371/journal.pmen.0000283)
Supplement: Attachment — Submitted filename: Parental Teasin Revision 2 Letter Aug.docx [file pmen.0000283.s003.docx]

Dear Dr. Karli Montague-Cardoso,

We are writing in response to the reviews of our manuscript, “Parental Teasing and Body Dissatisfaction in White and South Asian Females: An Exploratory Cross-Sectional Analysis Using Moderated Mediation,” submitted to *PLOS Mental Health*. We appreciate you taking over the handling of this manuscript and we sincerely value the insightful comments and constructive feedback provided by the second reviewer.

We have carefully addressed Reviewer 2’s suggestions and believe these revisions have strengthened our manuscript. Below, we provide a detailed point-by-point response to each of Reviewer 2’s comments, outlining the specific revisions made and indicating corresponding sections and pages of the revised manuscript. Again, we are grateful for both reviewers’ insightful comments and look forward to your response.

***Reviewer #1:***

1. *This is much improved and now suitable for publication in my view.*

Thank you for your previous review and your assistance is strengthening this manuscript.

***Reviewer #2:***

***General:***

1. *I strongly recommend replacing the word ‘Caucasian’ with ‘White’. Caucausian has some racist undertones and despite it being used in other publications, I strongly recommend not to perpetuate this (see e.g.,* [*https://www.careharder.com/blog/is-the-word-caucasian-racist*](https://www.careharder.com/blog/is-the-word-caucasian-racist)*).*

You are absolutely correct and thank you for raising this. We replaced the word “Caucasian” with “White” throughout the manuscript.

2. *Throughout the manuscript, you are switching between “depressive symptoms” and “depression” – I suggest using “depressive symptoms” throughout.*

Thank you for this recommendation. We now use the term “depressive symptoms” consistently throughout the manuscript.

3. *I have some concerns about your results in relation to the variable self-esteem. When looking at your correlation table, it seems like higher self-esteem is associated with higher body dissatisfaction, higher BMI, higher parental weight-related teasing and more depressive symptoms. Please check the coding, this might also explain your unexpected results of the moderated mediations.*

We appreciate this recommendation and have carefully revisited the coding for our self-esteem variable given our counter-intuitive results. Specifically, we re-coded results from the Rosenberg Self-Esteem Scale paying specific attention to items in the scale that required reverse coding. The values were the same as those in our original dataset and analyses with the re-coded values yielded identical results. Given these checks, we are confident that the findings reported in the manuscript reflect genuine relationships in our sample rather than methodological error.

Introduction

4. *“Additionally, Webb et al. (2020) reported that parental weight-related teasing during early adolescence was associated with emotional eating and increased over time” – What do you mean by "over time"? Do you mean the association became stronger in older adolescents, or are you referring to findings from a longitudinal study?*

Thank you for the opportunity to clarify. In Webb et al. (2020), ‘over time’ refers to findings from a longitudinal study in which the same participants were assessed one year apart. The authors found that parental weight-related teasing at Time 1 (Grade 5) prospectively predicted increased emotional eating at Time 2 (Grade 6), controlling for baseline levels. Thus, the association refers to a temporal change observed within individuals over time, not merely differences across age groups. We have clarified this point on page 4 of the revised manuscript.

5. *“The current study examined the cross-sectional association between parental teasing and BD South Asian minorities living in Westernized countries” IN Sout Asian minorities.*

Thank you we have corrected this typo in the revised manuscript.

6. *Some restructuring of the last 4 paragraphs of your introduction is needed- provide evidence from other studies first before starting to introduce your own study.*

Thank you for this recommendation. We have restructured the last 4 paragraphs of our introduction to provide a clearer delineation between our literature review and the introduction of the current study (pages 4-6).

7. *The presentation of your hypotheses could be clarified- no all hypotheses are consistent with a moderated mediation framework, I suggest the following: “As such, we aimed to address the following research questions using a moderated mediation framework: (1) Do self-reported levels of parental weight related teasing differ between Caucasian and South Asian participants? (2) Is parental teasing associated with BD, and is this relationship mediated by depressive symptoms? and (3) Does self-esteem moderate associations between teasing, depressive symptoms, and BD, consistent with a moderated mediation framework?”*

Thank you, we have revised our hypotheses to be consistent with your recommendations above.

8. *The associations between body dissatisfaction and weight-related teasing are introduced well in the introduction. However, there is very little literature cited that specifically addresses the relationships between weight-related teasing, depressive symptoms, and self-esteem—despite the fact that the moderated mediation models focus explicitly on these variables. Consider strengthening the introduction by citing relevant studies to justify the focus on self-esteem and depressive symptoms in the models.*

Thank you for this suggestion. We have revised the introduction to include a more detailed review of the literature addressing the associations between weight-related teasing, depressive symptoms, and self-esteem. Specifically, we now reference and summarize findings from several key studies that highlight the psychological sequelae of teasing, including Eisenberg et al. (2003), Haines et al. (2006), Neumark-Sztainer et al. (2002), and Quick et al. (2013). These studies demonstrate that weight-related teasing from family members is associated with increased depressive symptoms, lower self-esteem, and disordered eating behaviors, and that self-esteem may play a mediating role in these pathways. We believe these additions more clearly justify the inclusion of depressive symptoms and self-esteem in our moderated mediation models. (See page 4 of the revised manuscript).

***Methods***

9. *“computer or laptop to complete the study online” – what about participants who completed the study on their phones?*

Thank you for noticing this omission, we added phones (and tablets) to the device list.

10. *Body dissatisfaction- “6-point Likert rating scale ranging from 1 (i.e., never) to 6 (i.e., always), with higher scores indicating more BD.” – should be HIGHER BD*

Thank you we have corrected this error.

11. *“Each item is scored using a 4-point Likert rating scale ranging from 1 (i.e., strongly agree) to 4 (i.e., strongly disagree) and summed to create a total score, with a higher score indicating more positive self-esteem.” – check the coding of this variable*

Thank you, please see our response to point #3 above. Furthermore, we have added additional information to this section to highlight the requirement for reverse coding of some items in the Rosenberg Self Esteem Scale (page 8)

***Variables***

12. *“BMI, calculated using self-reported height and weight (kg/m²), was considered a potential confounder or covariate.” -call it a covariate, not confounder*

You are absolutely correct, we have removed the word “confounder”.

***Results***

13. *Immigrant status has not been described in the methods section*

The inclusion of immigrant status in the demographics questionnaire is mentioned within the first paragraph of the Procedures section within the Methods (page 7).

14. *Tests of mediation – italicize letters when reporting results*

Thank you, we have corrected this formatting error in the results section.

15. *“Given the cross-sectional nature of the data, however, causal interpretations should be viewed with caution, as reverse causation remains a possibility.” – since this is a cross-sectional dataset, causal interpretations cannot be made at all*

You are correct, we have removed this sentence from the revised manuscript.

***Tests of moderated mediation***

16. *I have concern about this analysis, as the sample size within self-esteem groups are extremely small. Even for exploratory purpose, these subgroups might be too small to support meaningful or reliable conclusions. I therefore suggest removing them from the manuscript.*

We appreciate the reviewer’s concern regarding subgroup sample sizes in our probing of the moderation effect. We respectfully note, however, that the grouping by self-esteem levels (high = 1 SD above the mean, moderate = mean, low = 1 SD below the mean) was not conducted as a primary stratified analysis, but as a widely recommended post hoc probing method to aid interpretation of continuous moderator effects in moderated mediation models (Aiken & West, 1991; Hayes, 2018). This approach is standard in PROCESS and related moderation frameworks and is intended to visualize and clarify significant interaction terms, rather than to produce independent statistical conclusions for each subgroup.

The subgroup n’s in our analysis (ranging from 22–34 in the smallest cells) are within the range considered adequate for probing interactions in moderation analyses in psychological research (e.g., Dawson, 2014; Hayes, 2018; Jose, 2013). Importantly, the moderation effects themselves were tested in the full sample using the continuous self-esteem variable, which preserves statistical power and minimizes the risk of Type II error (MacCallum et al., 2002). The plotted subgroup slopes simply illustrate the nature of these effects and follow accepted reporting conventions in the field.

Moreover, our moderated mediation findings are consistent with prior work suggesting that self-esteem may differentially shape the impact of interpersonal stressors such as teasing on psychological outcomes (e.g., Quick et al., 2013; Zeigler-Hill, 2011), thereby strengthening the theoretical rationale for their inclusion. Removing the probing results would limit readers’ ability to understand the practical meaning of the significant interaction terms, particularly given that our indirect effects were only supported at certain levels of self-esteem.

For these reasons, we believe retaining the subgroup probing results will provide important context for understanding the moderation effects and align with best practices in reporting moderated mediation models.

17. *Table 1 – make note more informative*

Thank you, we have revised the Table 1 note to indicate the following: “Table presents self-reported caregiver structure and immigration status for participants and their childhood caregivers, separately by ethnocultural group. “Caregiver immigrant status” indicates whether one or both primary caregivers were born outside of Canada. “Participant immigrant status” indicates whether the participant themselves was born outside of Canada. p*-*values reflect results of t-tests comparing frequencies between groups.”

18. *Table 2- add description of reliability score (Cronbach’s alpha) below the table*

Thank you, we have revised the note below Table 2 to include a description of Cronbach’s alpha as a measure of internal consistency reliability.

19. *Tables 3 and 4- combine this information into 1 table with one participant group above, the other one below the diagonal*

What an excellent recommendation! We have created a single Table (Table 3 in the revised manuscript) that includes both participant groups.

***Discussion***

20. *“This counterintuitive finding suggests that high self-esteem among Caucasian females may not provide a protective buffer against the negative impacts of weight teasing; instead, it may heighten the sensitivity to such teasing, possibly due to a stronger investment in body image ideals.” – these unexpected findings need to be discussed in more detail (after checking the self-esteem variable). Why do you think that those with higher self-esteem would be more invested in body image ideals?*

We agree that the observed positive association between self-esteem and body dissatisfaction in the White subgroup is counterintuitive and merits further discussion. Our revised discussion now addresses this explicitly by considering the possibility that, for some individuals, self-esteem may be contingent on meeting culturally dominant appearance standards (see Crocker & Wolfe, 2001; Crocker & Park, 2004). In such cases, high global self-esteem scores may reflect confidence that is heavily appearance-based, which could heighten sensitivity to body-related teasing or perceived deviations from the thin-ideal (Grabe et al., 2008). This may help explain why, in our White sample, higher self-esteem was associated with greater body dissatisfaction and greater reactivity to weight-related teasing. In contrast, the absence of this pattern among South Asian females could indicate less appearance-contingency in self-esteem or protective cultural factors that alter the self-esteem–body dissatisfaction link (Liang & Fassinger, 2008; Goel et al., 2021).

21. *Limitations- it should be noted that parental weight-related teasing was recalled retrospectively, while BD, self-esteem and depressive symptoms were assessed concurrently. This introduces the possibility that participants' current levels of BD, self-esteem or depressive symptoms may have biased their recollections of teasing experienced between the ages of 5 and 16.*

Thank you for this thoughtful observation. We have incorporated this point into the limitations section of the manuscript. Specifically, we now note that the retrospective assessment of parental weight-related teasing, alongside the concurrent measurement of BD, self-esteem, and depressive symptoms, introduces the possibility of recall bias. We acknowledge that participants’ current psychological states may have influenced their recollections of past teasing, which could inflate the strength of observed associations. (See page 17 of the revised manuscript).
